# Prevention of PVDF ultrafiltration membrane fouling by coating MnO_2_ nanoparticles with ozonation

**DOI:** 10.1038/srep30144

**Published:** 2016-07-20

**Authors:** Wenzheng Yu, Matthew Brown, Nigel. J. D. Graham

**Affiliations:** 1 Department of Civil and Environmental Engineering, Imperial College London, South Kensington Campus, London SW7 2AZ, UK

## Abstract

Pre-treatment is normally required to reduce or control the fouling of ultrafiltration (UF) membranes in drinking water treatment process. Current pre-treatment methods, such as coagulation, are only partially effective to prevent long-term fouling. Since biological activities are a major contributor to accumulated fouling, the application of an oxidation/disinfection step can be an effective complement to coagulation. In this study, a novel pre-treatment method has been evaluated at laboratory scale consisting of the addition of low dose ozone into the UF membrane tank after coagulation and the use of a hollow-fibre membrane coated with/without MnO_2_ nanoparticles over a test period of 70 days. The results showed that there was minimal fouling of the MnO_2_ coated membrane (0.5 kPa for 70 days), while the uncoated membrane experienced both reversible and irreversible fouling. The difference was attributed to the greatly reduced presence of bacteria and organic matter because of the catalytic decomposition of ozone to hydroxyl radicals and increase of the hydrophilicity of the membrane surface. In particular, the MnO_2_ coated membrane had a much thinner cake layer, with significantly less polysaccharides and proteins, and much less accumulated organic matter within the membrane pores.

Membrane fouling remains a major barrier limiting the application of ultrafiltration in water and wastewater treatment as a result of the increased operating costs and operational constraints (e.g. lower membrane fluxes). In particular, biological effects are a major component of fouling in many membrane-based separation processes. Biopolymers have been suggested to play a role in the fouling process, not only in membrane bioreactor (MBR) units^1^, but also in membrane systems in drinking water treatment^2^. It has been reported that proteins and polysaccharides could be the cause of biofouling^3^,^4^, as it improves the particles connected to membrane surface^5^.

Pre-treatment involving traditional chemical coagulation has been shown to be an effective and low-cost approach for controlling membrane fouling^2^,^6^,^7^, as well as improving water quality in general. However, unseparated flocs after the coagulation process typically collect at the surface of the membrane and form a cake layer containing many bacteria, which together with their associated extracellular polymeric substances (EPS), can lead to both reversible and irreversible membrane fouling.

Therefore, to prevent such fouling it is necessary to substantially decrease the ability of flocs (and bacteria) to attach or accumulated at the surface of the membrane. The connection/attachment ability is to some extent determined by the EPS concentration^8^, which in turn is determined by the bacterial activity. Some oxidative agents, such as chlorine, peroxide, potassium permanganate (KMnO_4_) and manganate (K_2_MnO_4_) are effective disinfectants and been found to have a beneficial effect on reducing fouling^8^,^9^,^10^. However, since these chemicals can also have negative impacts in the context of drinking water treatment (e.g. halogenated by-products from chlorination), and still can not mitigate membrane fouling fully.

When ozone has been applied as a pretreatment the results generally indicate a reduction in membrane fouling^11^,^12^,^13^, in cases where ozonation caused a significant degradation of influent biopolymers and/or colloidal natural organic matter (NOM)^14^,^15^. In full-scale trials ozone addition was reported to reduce biopolymer retention by UF membranes by up to 23%^16^. Membrane fouling was reported to be effectively retarded by ozonation in the long-term operation of MBRs^17^, and ceramic membrane fouling could be mitigated by ozonation combined with hydrogen peroxide in the treatment of raw waters^18^,^19^.

Previous studies have also considered modifying the membrane itself in order to minimize or prevent fouling, while current state of the art methods involve modification of the membranes with either hydrophilic additives or with an antibacterial compound^20^,^21^. Surface modification of membrane may enhance membrane performance through an anti-fouling process, such as polydopamine^22^,^23^. Coating nanoparticles on the membrane surface has been found to modify the membrane surface and improve its fouling resistance properties, such as by coating silver nanoparticles (AgNPs)^24^, SiO_2_ nanoparticles^25^,^26^ and TiO_2_ nanoparticles^27^,^28^,^29^,^30^. Also, a hybrid ceramic membrane process with iron nanoparticles effectively removed and transformed relatively high contents of aromatic, high molecular weight and hydrophobic natural organic matter (NOM) fractions^31^.

Coating nanoparticles on the surface of a membrane can change its properties by altering its hydrophilic nature^32^, its hydraulic resistance, and surface charge, such as Al_2_O_3_^33^. In general, an increase in hydrophilicity leads to reduced membrane fouling, and for polyvinylidene fluoride (PVDF) membranes this can be achieved through the addition of Ag/TiO_2_ nanoparticles because of the contribution of surface hydroxyl groups (OH)^34^. Gohari *et al*. synthesized and incorporated hydrous manganese dioxide (HMO) nanoparticles with polyethersulfone (PES) to fabricate nano-composite mixed matrix membranes (MMMs) for UF, and the membrane flux recovery was considerably enhanced^35^. Also the use of MnO_2_ as the basis of the membrane and found it was capable of a high containment removal efficiency^36^.

However, some researchers have found that long-term nano-silver exposure did not change the membrane fouling rate and even the concentration of extracellular polymeric substances (EPS) increased significantly after nano-silver dosing^37^. Therefore, it may need combine this method with others. Combining ozonation with other pre-treatment methods can enhance pollutant removal and mitigate membrane fouling, such as ozone with iron oxide nanoparticles^31^,^38^, because of the generation of hydroxyl radicals from the catalytic decomposition of ozone. Alternatively, ozonation-biological activated carbon (BAC) filtration can mitigate UF membrane fouling for treating activated sludge (AS) effluent^39^, and ozonation-powdered activated carbon (PAC) can remove aromatic DOC and other organic matter in drinking water treatment^40^. Manganese-catalyzed ozonation has been shown to be an advanced oxidation process through the generation of ·OH radicals^41^. The use of a manganese oxide surface layer as a catalyst for the oxidation of suspended and dissolved organic carbon in a combined ozonation-membrane filtration system treating natural water has been investigated^42^. The performance of the manganese oxide coated membrane was superior to that of the other membranes tested, showing the fastest recovery in permeate flux when ozone was applied and the greatest reduction in the total organic carbon (TOC)^43^, but until now, there is no research combining these methods with coagulation process. PVDF hollow fiber membranes have a high ozone resistance and should have life-times exceeding 5 years at low ozone doses^44^, so they are suitable for applications involving ozone pre-treatment.

In this paper we have evaluated the effectiveness of combining a UF membrane coated with MnO_2_ nanoparticles, with pre-treatment by coagulation and ozone oxidation in the membrane tank, for the treatment of raw water over an extended period of operation. The potential benefits of this novel process that have been investigated are a decrease in membrane fouling and higher permeate water quality, arising from the following mechanisms: 1) an increase in the hydrophilicity of the membrane surface because of the MnO_2_ coating; 2) catalytic reactions between the ozone and MnO_2_ nanoparticles generating ·OH radicals, recycle of Mn valence and enhanced degradation of organic substrates; 3) reduced bacteria and EPS concentrations within the membrane tank; 4) decrease bacteria attachment on the surface because of MnO_2_ and thus thinner cake layer.

## Experimental Methods and Materials

### Model raw water and coagulant

A model raw water was used for the tests in order to simplify the study and provide sample consistency and reproducibility. A quantity of wastewater effluent (Mogden Sewage Treatment Works, Thames Water, London, United Kingdom) was added to the local (London, United Kingdom) tap water with a volumetric ratio of 1:50, together with an amount equivalent to 5 mg/L Suwannee River Humic Acid (2S101H, International Humic Acid Substance Society, USA), to produce a model water representive of a lowland river. The effluent provides bacteria representative of surface waters contaminated by microorganisms from effluent discharges. Prior to mixing with the wastewater effluent and humic acid solution, the tap water was left over night to ensure the complete decay of residual chlorine. The characteristics of the model raw water are listed in Table 1. During the course of the experimental programme the temperature of the water was maintained at 21 ± 1 °C.

### Coating MnO_2_ nanoparticles on membrane

The membranes used in all tests were polyvinylidene fluoride (PVDF) hollow-fiber UF membrane module (Tianjin Motimo Membrane Technology Co., Ltd, China) with a nominal pore size of 0.03 μm and a surface area of 0.025 m^2^ (inner diameter = 0.7 mm, and outer diameter = 1.1 mm). The outer side of the membrane was coated with *in-situ* MnO_2_ nanoparticles, of 25 nm mean particle size, by dip-coating the membrane with a 21.7 wt% MnO_2_ solution prepared using the following procedure. Initially, a MnO_2_ nanoparticles suspension was created by mixing 0.5 L KMnO_4_ (2 mol/L) and 0.5 L MnCl_2_ (3 mol/L) together. The membrane fibres were then dipped vertically into the MnO_2_ nanoparticles suspension, and retained there for 1 hour with slow mixing. After this, the membrane module was removed and put into DI water with 1 min ultrasound (0.8 W/m^2^, KC3, 85W, KERRY, GUTSON, United Kingdom) to remove any loosely bound MnO_2_ before placing the membrane into the membrane tank directly. The membranes in the module were coated by MnO_2_ nanoparticles with a specific mass of around 200 mg/m^2^; this was quantified by an extended period (>10 min) and higher intensity (1.5 W/m^2^) of ultrasonication (Sonorex Digitec DT31, 160 W, Monmouth Scientific, United Kingdom) to release the nanoparticles, freeze-drying and weighing.

### The UF treatment systems

A schematic illustration of the experimental set-up involving the two coagulation-UF (CUF) processes, without and with a MnO_2_ layer (CUF-O_3_ and CUF-MnO_2_-O_3_, respectively) on the membrane (dead end mode), operated in parallel, is given in Figure S1 in Supporting information. Model raw water after coagulated with alum passed directly to the membrane tanks. Ozone was added in gaseous form (generated from air by ozone generator; KRC Marine Ltd, UK) at the bottom of the membrane module (Figure S1) at an applied dose of 1.0 mgO_3_/L. The gas flow rate was 0.5 L/min and a gas-phase ozone monitor (MP, ANSEROS, Germany) was used to measure the ozone concentration from the generator to the membrane tank and in the off-gas from the tank. From the difference of these the ozone consumed was approximately 0.36 mg/L corresponding to the applied does of 1.0 mgO_3_/L dose, respectively. Aqueous residual ozone concentrations in the membrane tank were below the level of detection using the indigo method^45^.

The UF permeate was continuously collected by a suction pump at a constant flux of 20 L/(m^2^ h), operated in a cycle of 30 min filtration and 1 min backwash (40 L.m^−2^.h^−1^ water and 100 L/h air in the membrane tank). The whole operation process lasted for 70 days. The trans-membrane pressure (TMP) was continuously monitored by pressure gauges. The HRT of the membrane tanks was maintained at 0.5 h and accumulated, settled sludge in each tank was released every day. During this period, the membrane only in the CUF-O_3_ system was taken out and washed by sponge at day 30.

### Extraction and measurements of EPS from cake layer and sludge

At the end of the UF operation, the foulant materials on the membrane surface (cake layer) were carefully scraped off with a plastic sheet, and analyzed by the following methods to characterize the contents. A heating and extraction method^46^ was used to extract the loosely bound EPS (LB-EPS) and tightly bound EPS (TB-EPS) from the cake layer and sludge, which is introduced in detail previously^8^. After the membrane surface was wiped with a sponge, 0.01 mol/L NaOH was used for extraction of internal foulants and the fibers were soaked for 24 h at 20 °C in the alkaline solution according to the method described by Kimura *et al*.^47^ and Liu *et al*.^48^. The extracted organic matter was then subjected to the following chemical analyses.

The absolute polysaccharide content in the bound EPS (LB-EPS plus TB-EPS) was measured by the phenol–sulfuric acid method with glucose as the standard^49^. A modification of the Bradford method^50^ called the Coomassie procedure (Pierce Chemical), was used to quantify the absolute concentration of proteins, with bovine serum albumin (Sigma) as the standard. EPS extracted from the cake layers and sludges were also analyzed by size exclusion chromatography (SEC), which is introduced in detail previously^8^.

### Other analytical methods

Fouled membrane fibers were cut from the two membrane modules, and the foulant layer attached on the membrane surface was retained on the membrane surface. The fouled membrane samples were then platinum-coated by a sputter and observed under high resolution field emission gun scanning electron microscope (FEGSEM, LEO Gemini 1525, Germany).

Thermogravimetric differential thermal analysis (TG-DTA) of water samples and sludges after freeze drying were carried out (TA Instruments, NEJSCH STA 449C), and the specific functional groups of organic matter in the raw water and effluents were analyzed by Fourier Transform Infrared spectroscopy (FTIR, Spectrum 400, PerkinElmer, USA) with Quest ATR Accessory (SPECAC Ltd, UK), also after freeze drying. The UV absorbance at 254 nm, UV_254_, of 0.45 μm filtered solutions was determined by an ultraviolet/visible spectrophotometer (U-3010, Hitachi High Technologies Co., Japan). Dissolved organic carbon (DOC) was determined with a total organic carbon (TOC) analyzer (TOC-V_CPH_, Shimadzu, Japan). Residual turbidity was determined by a commercial turbidimeter (Hach 2100, USA). The concentrations of 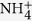-N and 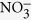-N were determined by the APHA standard colorimetric/spectrometry methods^51^, and the concentrations of bacteria were quantified as the Heterotrophic Plate Count (HPC) by the recommended method involving the use of yeast extract agar^52^.

Hydrophilic and hydrophobic organic components were evaluated as follows: Superlite DAX-8 (Supelco, USA) and Amberlite XAD-4 (Rohm and Hass, Germany) resins were used to fractionate NOM into three groups: strongly hydrophobic organic matter (adsorbed by DAX-8), weakly hydrophobic organic matter (adsorbed by XAD-4) and hydrophilic organic matter (fraction passing through both resins)^53^,^54^.

## Results

### TMP developments in CUF-O_3_ and CUF-MnO_2_-O_3_ systems

**Figure 1:**
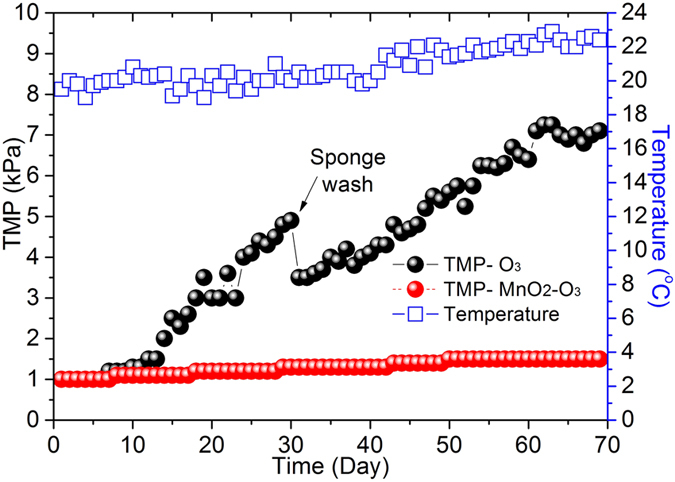
Variation of TMP with time and pretreatment conditions over a period of approximately 70 days. (Membrane flux was maintained at a constant value (20 L/m^2^h), and membrane only in the CUF-O_3_ system was taken out and washed by sponge at day 30).

The comparative increase in TMP for the CUF-O_3_ and CUF-MnO_2_-O_3_ streams are shown in Fig. 1, which could represent membrane fouling as membrane flux was maintained at a constant value. There was no detectible, additional pressure loss caused by MnO_2_ nanoparticles coating on the membrane in the CUF-MnO_2_-O_3_ system at the start of process operation, as expected at such a low filtration rate. Comparing the two types of the pretreatment, the presence of the MnO_2_ surface coating resulted in a membrane fouling rate that was substantially lower than that without coating. During the initial 10 days of operation the increase in TMP of the both membranes was very low, partly as a consequence of the presence of ozone limiting the development of biological activities and bio-accumulation on the membranes. Subsequently, there was a steady increase of TMP in the CUF-O_3_ system up to 5 kPa at 30 days (approximately 0.2 kPa/d), while virtually no increase in the CUF-MnO_2_-O_3_ system over the same period. Thus, at day 30 when the CUF-O_3_ membrane was taken out and washed by sponge, the TMP increase of the CUF-MnO_2_-O_3_ membrane was <0.5 kPa, indicating the significance and benefit of the MnO_2_ coating layer as a membrane protection.

After physical cleaning of the CUF-O_3_ membrane (membrane was taken out and cleaned by high pressure tap water and sponge) at day 30, a greater initial TMP was found (3.2 kPa) compared to the original membrane, which may be related to the difficulty of removing organic contaminants in the CUF-O_3_ membrane pores, as well as some residual cake layer on the surface of the membrane. In marked contrast, the increase in TMP of the CUF-MnO_2_-O_3_ membrane over the full 70 days of operation was only 1.5 kPa. In this case it was assumed that the low membrane fouling was caused by the gradual accumulation of oxidized organic material resistant to further degradation by ozone and MnO_2_-catalyzed ·OH radicals, either in the surface cake layer or in the membrane pores, or both. Also, it is believed that the characteristics of the cake layer on the surface of membrane are sufficiently altered by the exposure to O_3_/·OH, as to enable a near-complete removal during the routine backwashing.

In summary, it was evident that the internal and external membrane fouling in the CUF-O_3_ process was much greater than the CUF-MnO_2_-O_3_ process. In view of the enhanced performance arising from the MnO_2_ surface coating, further detailed investigation was undertaken concerning the nature of the membrane with and without MnO_2_ nanoparticles, including the organic matter and the extent of bacterial activity in the two membrane systems.

### Characteristic of organic matter and bacteria concentration in membrane tanks

**Figure 2:**
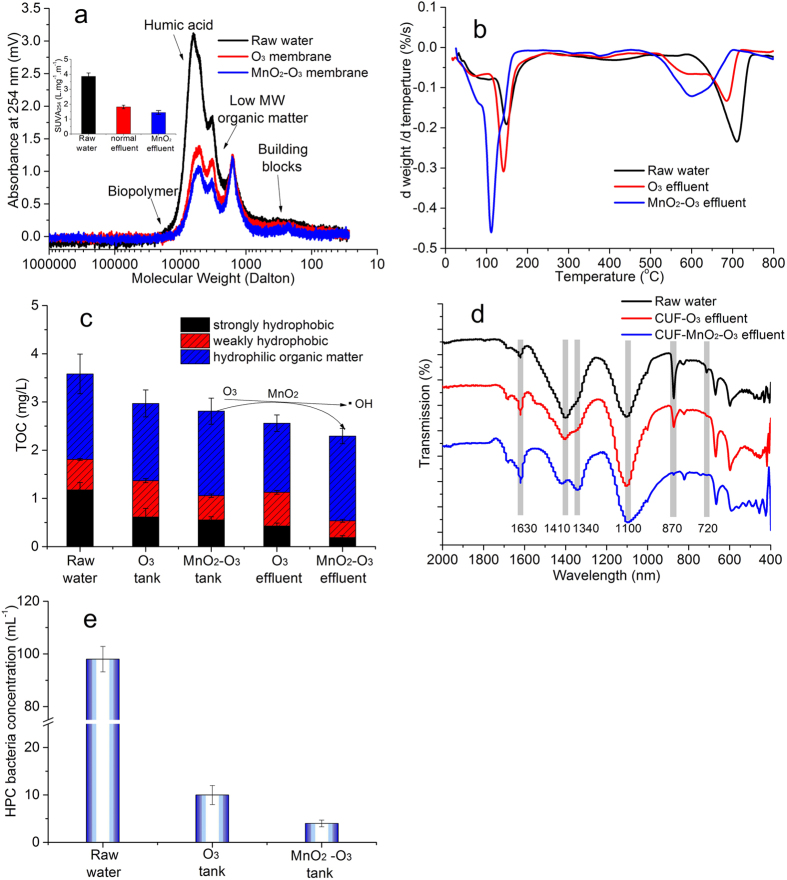
Comparative properties of organic matter and HPC bacteria concentration (samples taken at day 50 or averaged for several times) for the CUF-O_3_ and CUF-MnO_2_-O_3_ systems: (**a**) molecular weight and SUVA of organic matter; (**b**) DSC-TGA (N_2_ gas); (**c**) FTIR spectra; (**d**) proportion of hydrophilic and hydrophobic components; (**e**) HPC bacteria concentrations (Water samples from membrane systems were collected for SEC and TOC measurement, and for DSC-TGA and FTIR measurement the water samples were freeze-dried. More organic matter was removed or transformed into smaller molecular weight in the CUF-MnO_2_-O_3_ system than that in the CUF-MnO_2_-O_3_ system).

The presence of organic matter and bacteria in the feed waters was evaluated as these influence membrane fouling. Organic matter was characterized by molecular weight (MW), thermogravimetric analysis (after freeze drying), FTIR and hydrophilic properties. Figure 2a shows that the MW distributions obtained from the SEC chromatograms displayed significant differences in the raw water and two membrane effluents, particularly in the range of the large molecules identified as biopolymers and humic acid. It can be seen that much of the organic matter was removed by the coagulation and then ozone oxidation processes, and more organic matter was removed in the MnO_2_-coated membrane system. Molecular ozone is known to be effective in attacking aromatic moieties via electrophilic addition, followed by ring cleavage^55^, and ·OH radicals can react indiscriminately, thereby enhancing the rate and extent of organic degradation. The SUVA value (specific UV absorbance), which is a proxy for dissolved organic matter (DOM) aromaticity^56^, was lower in CUF-MnO_2_-O_3_ permeate compared to that of the CUF-O_3_, confirming the greater oxidation associated with the catalytic action of the MnO_2_ coating.

Also, the organic matter in the membrane effluents (permeates) and raw water was characterized (after freeze drying) by thermogravimetric (TG-DTA) analysis (Fig. 2b). The TG-DTA analysis revealed that for all the samples the principal weight loss occurred at temperatures in the ranges of 50~200 °C and 500~800 °C. In the lower temperature range (50~200 °C), the weight loss of the NOM from the raw water was much less than from the effluents, and the peak loss was at a higher temperature (~160 °C). In comparison, the effluent of the CUF-MnO_2_-O_3_ system had the greatest loss of weight and at the lowest temperature (~110 °C). The corresponding results for the CUF-O_3_ effluent were between those of the raw water and CUF-MnO_2_-O_3_ effluent (i.e. peak weight loss temperature ~145 °C). The corresponding behavior observed at the higher temperature range of 500–800 °C was similar to that at 50~200 °C in terms of the comparative temperature at peak weight loss (i.e. lowest for CUF-MnO_2_-O_3_ effluent, greatest for raw water), but the weight loss was the greatest for the raw water and least for the CUF-MnO_2_-O_3_ effluent. These results are consistent with those in Fig. 2a indicating the removal and conversion of large MW organic matter into smaller MW by the combination of coagulation and oxidization, with the greatest effects occurring in the system with the MnO_2_–coated membrane due to the greater oxidation by MnO_2_–catalyzed ·OH radicals.

Besides the MW and concentrations of organic matter, its hydrophilic and hydrophobic properties are also of importance (Fig. 2c), as membrane fouling increases with hydrophobicity of the organic matter by adsorption on to the PVDF membrane and pores during the long period of membrane operation. The results show that coagulation and ozonation removed some of the organic matter from the raw water (see comparative values in the membrane tank, Fig. 2c), and much of the strongly hydrophobic organic matter was changed into either weakly hydrophobic or hydrophilic fractions. Comparing the two systems it is clear that more hydrophobic organic matter was converted into hydrophilic matter in the CUF-MnO_2_-O_3_ system, and especially when comparing the distribution of organic fractions in the respective effluents. Comparing the water in the membrane tank with the effluent for the MnO_2_-O_3_ system, a substantial proportion of the hydrophobic organic matter was converted into hydrophilic organic matter (nearly 50%), most likely as a consequence of the catalytic oxidation (O_3_/MnO_2_) on the surface of the membrane. As hydrophobic organic matter is much easier to be adsorbed onto the hydrophobic PVDF membrane pores, the conversion of hydrophobic organic compounds to hydrophilic should mitigate inner membrane fouling, especially for the MnO_2_-O_3_ system with the greater oxidation conditions at the membrane surface.

The comparative FTIR spectra of the organic matter in the feed water and membrane effluents can be seen in Fig. 2d. Comparing the effluent spectra with the raw water, the peak at 1630 cm^−1^ (C=O) increased as a consequence of the oxidation, especially in the MnO_2_-O_3_ system, which indicated that alcohol or carboxylic acid groups are formed during the processes. These results are consistent with previous studies showing that there is an increase in electron withdrawing groups after ozone treatment of aqueous organic matter^31^. Furthermore, a spectral band at 1340 cm^−1^ (C–H) became more distinct in both permeates, especially in the MnO_2_ coated membrane system, and this corresponded with the decrease of the peak at 1410 cm^−1^. A decrease in the band intensity at around 1410 cm^−1^, 870 cm^−1^ and 720 cm^−1^, especially in the MnO_2_-O_3_ system, indicated that the C–O stretching of esters, ethers, and phenols (1410 cm^−1^), and C–C anti-symmetric ring stretching of epoxides (870 cm^−1^ and 720 cm^−1^), decreased^57^.

As well as the fouling of UF membranes by organic matter, bio-fouling may also be an important phenomena, and arises from the presence of an active microbial community^58^. Therefore, the presence of viable bacteria (HPC) in the membrane tanks was investigated for the two systems during their operation and was found to be at much lower levels than the raw water (Fig. 2e), indicating that bio-foulng may not be a major effect. Furthermore, the bacteria concentration in the CUF-MnO_2_-O_3_ system (4 mL^−1^) was significantly less than the CUF-O_3_ system (10 mL^−1^), suggesting that the reduced fouling observed in the former system was partly attributed to a reduced level of bio-fouling.

### EPS in the cake layer and sludge

**Figure 3:**
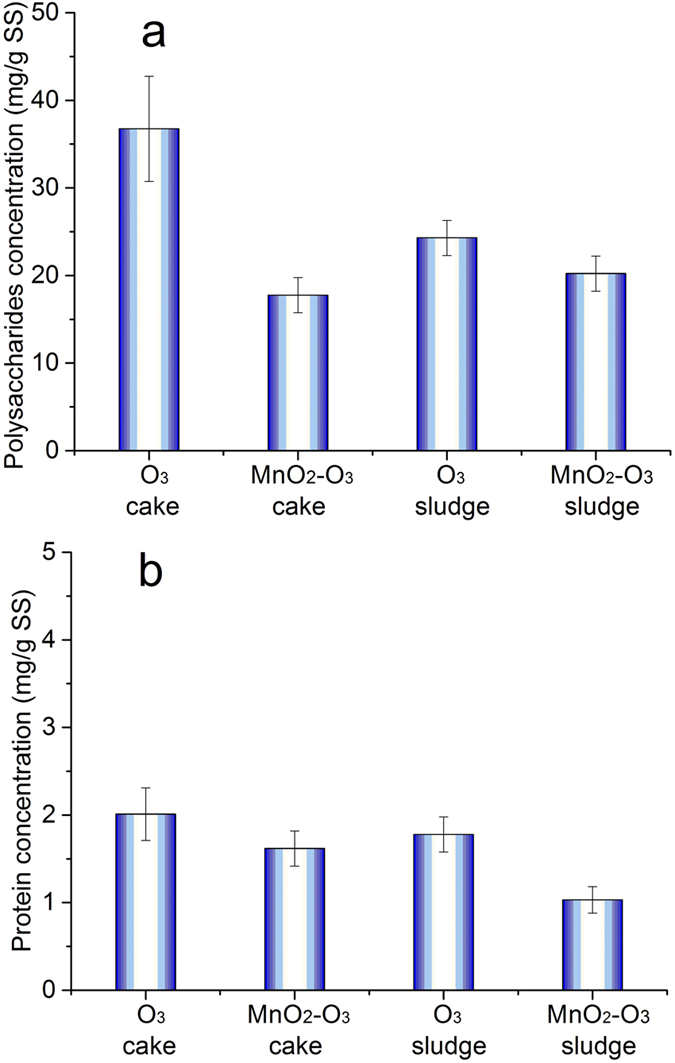
The EPS concentration in the cake layers and sludges for the two membrane systems at day 69: polysaccharide (**a**) and protein (**b**) (Polysaccharide and protein concentration were higher in the CUF-O_3_ membrane cake layer than CUF-MnO_2_-O_3_ cake layer).

The absolute EPS concentration in the cake layer and sludge of the two systems was investigated (Fig. 3). For the cake layer of the two systems, the polysaccharide concentration in the CUF-O_3_ cake layer was much higher than the CUF-MnO_2_-O_3_ (nearly two times, 0.036 g/g SS and 0.018 g/g SS) (Fig. 3a). However, the polysaccharide concentration in the CUF-O_3_ sludge was only slightly greater than the CUF-MnO_2_-O_3_ sludge (viz. 0.022 g/g SS and 0.020 g/g SS, respectively), which suggested that while polysaccharide concentrations in the CUF-O_3_ cake could accumulate with time, this was less likely in the case of the MnO_2_ coated membrane owing to the greater oxidizing conditions and greater polysaccharide degradation. A contributing factor also was the lower concentration of bacteria in the CUF-MnO_2_-O_3_ tank (Fig. 2e), which most likely produced less polysaccharide concentration. The corresponding results for protein showed that concentrations were much lower in the two membrane tanks compared to polysaccharides, and the existence of the MnO_2_ catalyst on the surface of membrane corresponded to a lower protein concentration. As the protein concentrations were approximately 10 times lower than the polysaccharides, it can be concluded that the much lower membrane fouling observed with the CUF-MnO_2_-O_3_ system was related mainly to the lower polysaccharide concentration in the cake layer; this layer was significantly thinner than the cake layer on the CUF-O_3_ membrane surface, as discussed later.

**Figure 4:**
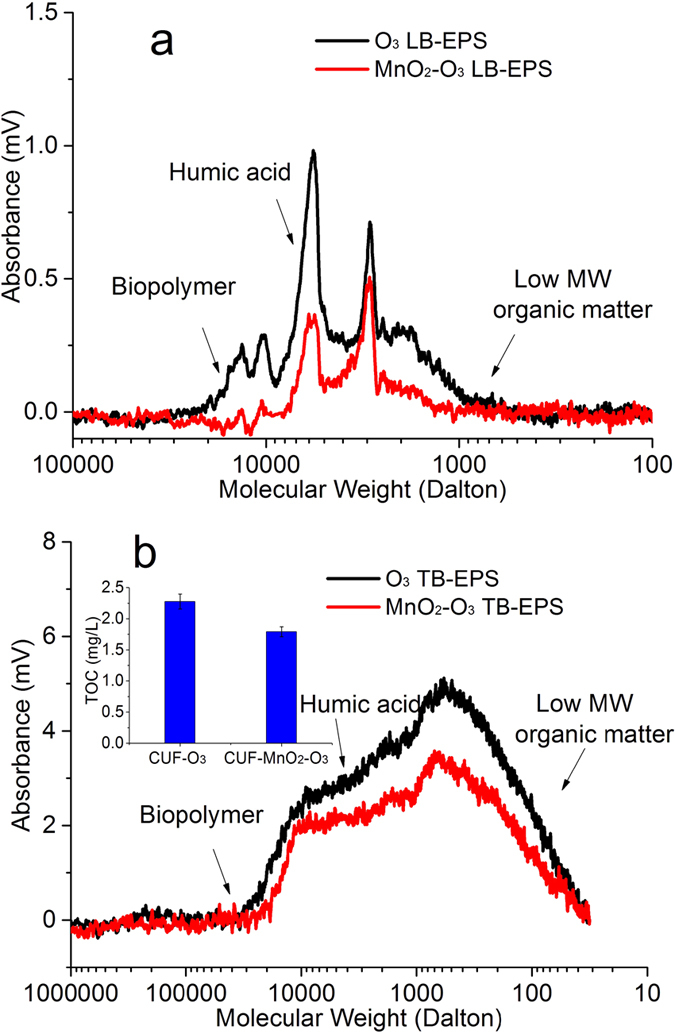
MW distributions of DOM from the CUF-O_3_ and CUF-MnO_2_-O_3_ cake layers: (**a**) LB-EPS, (**b**) TB-EPS (Both species of EPS were much higher in the CUF-O_3_ cake layer than that of CUF-MnO_2_-O_3_).

The MW results from SEC analysis were also used to characterize the EPS from the CUF-O_3_ and CUF-MnO_2_-O_3_ cake layers (Fig. 4). These show clearly that the quantity of LB-EPS biopolymers (10^5^~10^4^) in the CUF-O_3_ cake layer was much greater than the CUF-MnO_2_-O_3_ cake layer, which is consistent with the reduced bacteria concentration (hence less biopolymers - EPS) in the CUF-MnO_2_-O_3_ membrane tank (Fig. 2e). In addition, the concentration of TB-EPS, for all MW compounds, was much greater in the cake layer of the CUF-O_3_ system than for the CUF-MnO_2_-O_3_ system (Fig. 4b). These results clearly indicated that EPS present in the raw water and produced by bacteria within each process was removed or oxidized much more in the CUF-MnO_2_-O_3_ system, and less was accumulated in its cake layer; this was further supported by the measured TOC concentrations. The existence of a greater concentration of EPS in the CUF-O_3_ cake layer, which may detach and approach the membrane pores, and cause internal, irreversible membrane fouling (Fig. 1). This result found is similar to other researchers^59^. The lower concentration of EPS in the CUF-MnO_2_-O_3_ membrane enables the cake layer to be more easily removed than the cake layer on the CUF-O_3_ membrane surface during the backwash process (as confirmed by the observations later).

### Characteristics of organic matter on the membrane

As described in section 2.3, the membrane fibers before and after operation were examined by ATR-FTIR spectroscopy to examine the membrane fouling. In addition, fouling material within the membranes were extracted by NaOH and analyzed by SEC, to explore the inner membrane fouling.

**Figure 5:**
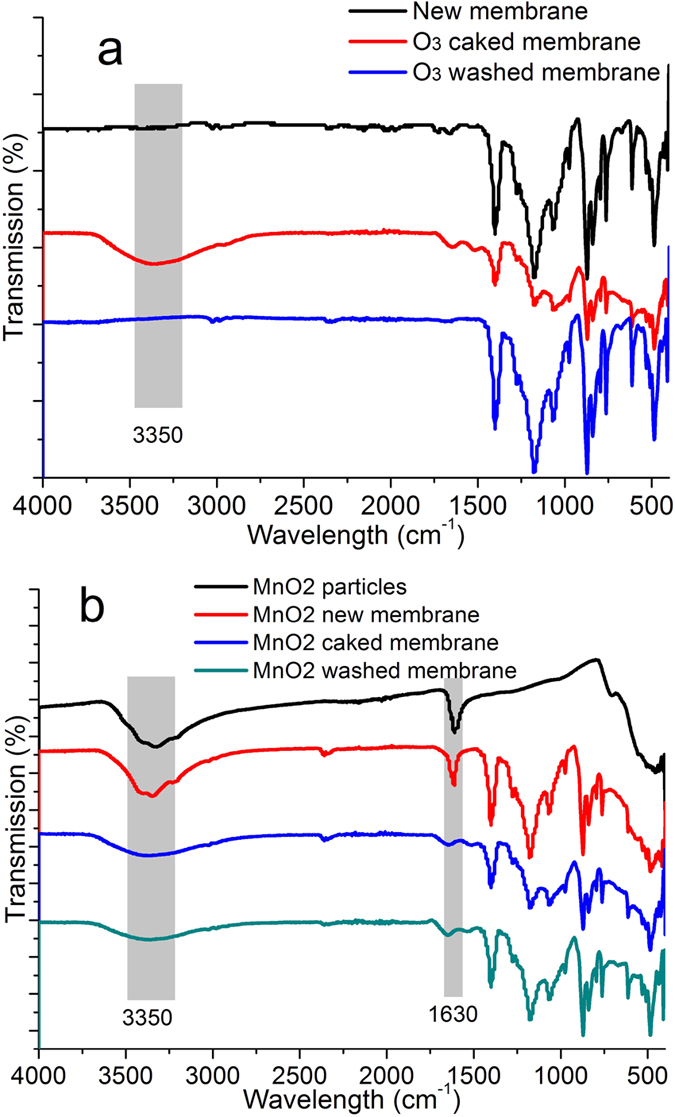
FTIR spectra for the CUF-O_3_ membrane system (**a**) and the CUF-MnO_2_-O_3_ membrane system (**b**) (FTIR spectra confirmed that there was much more cake layer accumulated on the CUF-O_3_ membrane surface).

The ATR-FTIR spectrum showed that there are lots of absorption peaks for the case of a new PVDF membrane (Fig. 5a). The peaks at 680, 763, 870, 1016, 1174, and 1400 cm^−1^ correspond to the CF_2_ and CH_2_ chemical bonds^60^, and there was no peak at around 3400 cm^−1^ (–OH peaks), which meant that the membrane was hydrophobic. After the CUF-O_3_ membrane was operated for nearly 70 days there was a peak at around 3400 cm^−1^, and the FTIR spectrum had broad overlapping bands instead of sharp absorption peaks, which was because of the cake layer on the surface of the membrane. Following washing of the cake layer and membrane, the FTIR spectrum was very similar to that of a new membrane, which meant that for the CUF-O_3_ membrane, the presence of residual, adsorbed organic matter was not very significant.

**Figure 6:**
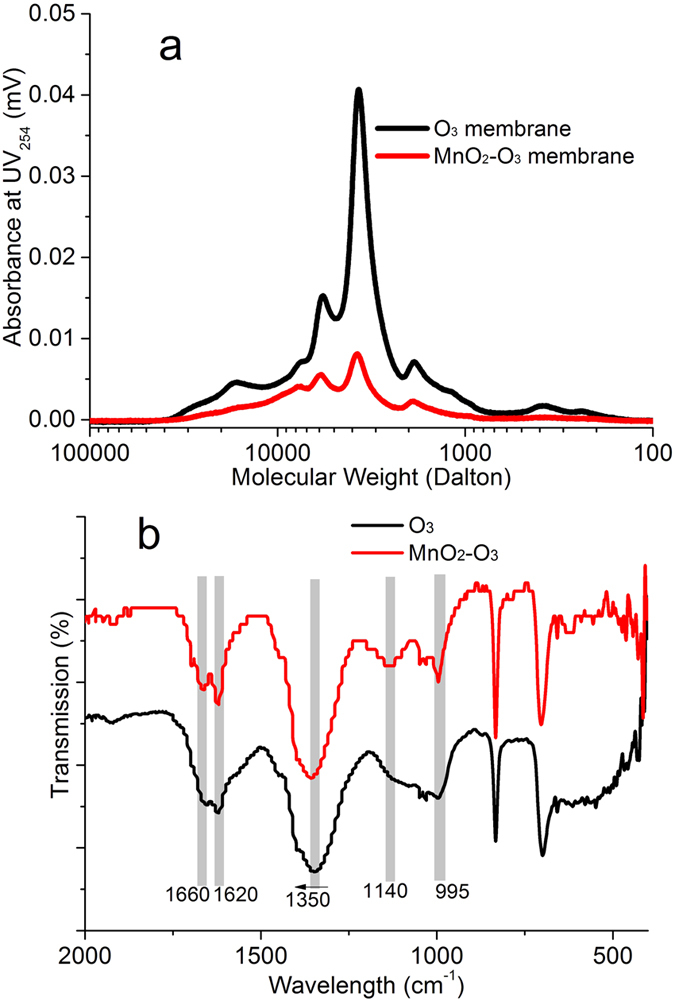
Analysis of DOM from inner membrane fouling in the two membrane systems: (**a**) MW distributions; (**b**) FTIR absorbance (DOM from inner membrane fouling in the two membrane systems extracted by 0.01 M NaOH, and then adjusted to pH 7 by 0.01 M HCl. There was much less organic matter adsorbed in the CUF-MnO_2_-O_3_ membrane after the systems were operated for nearly 70 days).

For the membrane coated with MnO_2_ nanoparticles (CUF-MnO_2_-O_3_), the FTIR spectrum included peaks corresponding to MnO_2_ particles and to a new MnO_2_ coated PVDF membrane (Fig. 5b). ATR/FTIR characterization confirmed that significant amounts of hydroxyl groups (OH) were formed on the PVDF membrane surface owing to the embedding of MnO_2_ (Fig. 6b). γ-MnO_2_ exhibits a clear absorption peak at 1620 cm^−1 ^61,62, which is usually associated with water of crystallization (around 3400 cm^−1^). This peak is assigned to the deformation of water molecules and indicated the presence of physisorbed water on the oxides^63^. After operation for 70 days, the FTIR spectra of the caked, and washed, CUF-MnO_2_-O_3_ membrane were nearly the same as an unused membrane, except for the peaks at 3400 cm^−1^ and 1630 cm^−1^, where the intensity had decreased compared to the new coated membrane. This was likely the result of some degree of detachment of MnO_2_ nanoparticles from the membrane surface at the start of membrane operation. Considering the spectra for the membranes with cake layers, the much sharper peaks evident in the CUF-MnO_2_-O_3_ system (Fig. 5b), compared to the membrane without MnO_2_, CUF-O_3_, indicating a greater thickness of the CUF-O_3_ cake layer.

In order to evaluate the nature of the organic matter that accumulated on to the surface, and within the pores, of the membranes at the end of operation (day 70), each membrane was soaked in 0.01 M NaOH solution and the extract analyzed. The SEC results clearly indicated that less organic matter was retained or adsorbed in the CUF-MnO_2_-O_3_ membrane pores, compared to the CUF-O_3_ system, with a lower concentration of biopolymer and humic-like materials (Fig. 6a). As described previously the MnO_2_ catalyzed decomposition of O_3_ to ·OH enhanced the oxidation conditions and induced a large reduction of biopolymers and other organic substances to lower MW, more hydrophilic species (e.g. with the –COO^−^ structure), leading to less organic matter adsorbed in the membrane pores. The greater concentration of EPS on the CUF-O_3_ membrane was consistent with its higher internal fouling resistance, indicated by the greater TMP development of the CUF-O_3_ system observed (Fig. 2).

The FTIR spectra of the NOM from membrane pores were also obtained (Fig. 6c). The organic matter from the two membrane systems were similar except at peaks 1620 cm^−1^, 1140 cm^−1^ and 995 cm^−1^, which were sharper or more distinct in the CUF-MnO_2_-O_3_ system. The effect of coating MnO_2_ nanoparticles on the surface of the membrane and associated oxidation conditions appeared to increase the formation or presence of particular chemical species. The absorption peak at 1620 cm^−1^ increased in the CUF-MnO_2_-O_3_ system, indicating that the oxidation led to the formation of alcohol or carboxylic acid groups. In addition, the absorption intensity at 1200~1000 cm^−1^, which corresponds to the C-O bond, and to the antisymmetric and symmetric stretching of ether bonds, appeared with the presence of MnO_2_ nanoparticles. Previous studies have indicated that coupling reactions between phenoxy radicals can lead to the formation of ethers^57^,^64^. The C–C bond at around 995 cm^−1^ increased, which indicates that some kind of alcohol or carboxylic acids increased more in CUF-MnO_2_-O_3_ system and membrane^31^.

### Fouled membrane structure by SEM images

**Figure 7:**
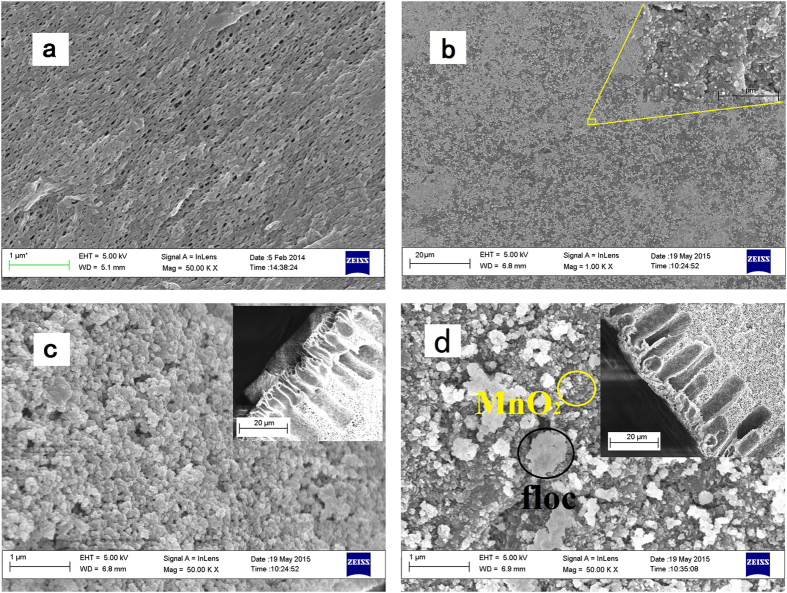
SEM images of new membranes with/without coating MnO_2_ and after operation for 70 days with pre-coagulation: (**a**) new membrane, (**b**) MnO_2_-coated new membrane, (**c**) fouled membrane in CUF-O_3_ system, (**d**) fouled membrane in CUF-MnO_2_-O_3_ system. (The cake layer on the CUF-O_3_ membrane surface consisted of thousands of nano-particles from flocs, while in the CUF-MnO_2_-O_3_ system, little cake layer formed by flocs could be seen on the membrane surface, and MnO_2_ particles could be seen on the membrane surface after operating for 70 days).

In order to support the results indicating that membrane fouling can be greatly mitigated by coating MnO_2_ nanoparticles onto the surface of the membrane, SEM images were obtained (Fig. 7). The new membrane surface displayed a high number density of large pores and the pore distribution appeared relatively uniform (Fig. 7a). When coated with newly formed MnO_2_, very large numbers of nanoparticles were evident on the surface of the membrane, with their size estimated to be around 5~10 nm (Fig. 7b).

After operation for 70 days, it was found that a continuous deposit layer of approximately 30 nm sized particles was present on the surface of the CUF-O_3_ membrane, which appeared to completely block the membrane pores (Fig. 7c). SEM images were also used to provide information about the thickness of the cake layer on the surface of the membranes; the cake layer was found to be much thicker on the CUF-O_3_ membrane surface than the CUF-MnO_2_-O_3_ membrane. The higher concentration (although generally low) of EPS in the cake layer is believed to induce greater external membrane fouling, partly by increasing the connection ability between nano-scale primary particles.

In marked contrast, there were substantially fewer residual flocs on the surface of the CUF-MnO_2_-O_3_ membrane. It is believed that the existence of MnO_2_ nanoparticles on the surface of the membrane reduces the possibility of bacteria attachment onto the surface of the membrane, with consequently less associated proteins and polysaccharides (EPS) present, and decreases the attachment of flocs on to the membrane. Owing to the thicker cake layer on the CUF-O_3_ membrane, and possibly a greater density, its hydraulic resistance was greater than the CUF-MnO_2_-O_3_ membrane after a long operation time, corresponding to the greater extent of external membrane fouling observed (Fig. 1).

In summary, the results indicated that during the period of operation, the interaction of dissolved ozone and the MnO_2_-coated membrane significantly reduced the presence of EPS, and little EPS passed through the membrane pores, causing less flocs to attach on the surface of membrane and much lower irreversible internal fouling. The superior performance is mainly explained by the enhanced oxidation conditions at the membrane surface with the MnO_2_ catalyzing the O_3_ decomposition to ·OH radicals.

## Conclusions

1. The presence of a MnO_2_ coating on the UF membrane had a major, and beneficial, impact on the development of internal and external membrane fouling. While for the CUF-O_3_ process there was a steady increase in TMP over the 70 days of operation, there was very little increase in TMP (~0.5 kPa) in the CUF-MnO_2_-O_3_ system.
2. While much of the influent organic matter was removed in the treatment processes by coagulation and ozone oxidation, more organic matter overall was removed by the CUF-MnO_2_-O_3_ system, and particularly the large MW organic substances appeared to be oxidized into more hydrophilic compounds, which mitigated inner membrane fouling of the hydrophobic PVDF membrane.
3. Coating MnO_2_ nanoparticles on the membrane surface was able to further decrease the presence of bacteria, which in turn reduced EPS concentrations, especially the polysaccharide concentration. During the operation period, oxidation by ozone at the surface of the MnO_2_-coated membrane significantly reduced the EPS concentration in the cake layer and little EPS passed through membrane pores; this resulted in fewer flocs attaching to the surface of the membrane and much lower irreversible internal fouling.
4. The successful prevention of membrane fouling in the CUF-MnO_2_-O_3_ system is attributed to the superior oxidation/disinfection conditions provided by the MnO_2_ nanoparticles, through the generation of ·OH radicals from ozone decomposition at the surface of the membrane.

## Additional Information

**How to cite this article**: Yu, W. *et al*. Prevention of PVDF ultrafiltration membrane fouling by coating MnO_2_ nanoparticles with ozonation. *Sci. Rep.* **6**, 30144; doi: 10.1038/srep30144 (2016).

## Supplementary Material

Supplementary Information

## Figures and Tables

**Table 1:** Quality of model raw water and UF filtrates^a^.

**Parameter**	**Raw water**	**CUF-O**_**3**_ **tank**	**CUF-MnO**_**2**_**-O**_**3**_ **tank**	**CUF-O**_**3**_ **filtrate**	**CUF-MnO**_**2**_**-O**_**3**_ **filtrate**
UV_254_(cm^−1^)	0.106 ± 0.017	0.041 ± 0.004	0.039 ± 0.003	0.038 ± 0.004	0.032 ± 0.002
DOC(mg/L)	3.51 ± 0.34	2.96 ± 0.11	2.78 ± 0.12	2.49 ± 0.14	2.29 ± 0.12
Turbidity(NTU)	2.04 ± 0.29	5.05 ± 0.33	4.95 ± 0.28	0.05 ± 0.01	0.04 ± 0.01
SS(mg/L)	3.5 ± 0.7	122.5 ± 11.2	126.0 ± 13.6	/	/
Zeta potential(mV)	−20.52 ± 1.32	−8.23 ± 0.35	−12.94 ± 0.47	/	/
pH	8.06 ± 0.06	7.90 ± 0.05	7.96 ± 0.04	8.05 ± 0.04	8.11 ± 0.04

^a^The values in Table 1 are averages (standard deviations) for all the measurements made every 7 days (9 times).
