# Supplementary material for: Prevention of PVDF ultrafiltration membrane fouling by coating MnO2 nanoparticles with ozonation
Source: Sci Rep. 2016 Jul 20;6:30144. doi: 10.1038/srep30144 (PMC4951810; doi:10.1038/srep30144)
Supplement: Supplementary Information [file srep30144-s1.doc]

**Prevention of PVDF ultrafiltration membrane fouling by coating MnO_2_ nanoparticles with ozonation**

Wenzheng Yu*, Matthew Brown and Nigel. J. D. Graham*

^a^ Department of Civil and Environmental Engineering, Imperial College London, South Kensington Campus, London SW7 2AZ, UK.

(w.yu@imperial.ac.uk, matthew.brown11@imperial.ac.uk, n.graham@imperial.ac.uk)

**Corresponding author: Tel: +44 2075946121, Fax: +44 2075945934*

**Supporting information**

**The UF treatment systems**

A schematic illustration of the experimental set-up involving the two coagulation-UF (CUF) processes, without and with a MnO_2_ layer (CUF-O_3_ and CUF-MnO_2_-O_3_, respectively) on the membrane (dead end mode), operated in parallel, is given in Figure S1. Model raw water was fed to a constant-level tank to maintain sufficient water head for the membrane tanks. An optimal dose of Al_2_(SO_4_)_3_ coagulant (0.15 mM, calculated as Al) was continuously added to the rapid mixing units; the alum dose corresponded to near zero zeta potential of resulting flocs. The rapid mix speed was 200 rpm (184 s^-1^) in the mixing units with a hydraulic retention time (HRT) of 1 min, which then reduced to 50 rpm (23 s^-1^) in the three flocculation tanks, each having a HRT of 5 min. From the flocculation tanks the flow passed directly to the membrane tanks. For the CUF-MnO_2_-O_3_ tank, the membranes in the module were coated by MnO_2_ nanoparticles with a specific mass of around 200 mg/m^2^. Ozone was added in gaseous form (generated from air by ozone generator; KRC Marine Ltd, UK) at the bottom of the membrane module (Figure S1) at an applied dose of 1.0 mgO_3_/L; the ozone dose was selected as lying at the lower end of the range typically used in water treatment practice. The gas flow rate was 0.5 L/min and a gas-phase ozone monitor (MP, ANSEROS, Germany) was used to measure the ozone concentration from the generator to the membrane tank and in the off-gas from the tank (Figure S1, points 21 and 22, respectively). From the difference of these the ozone consumed was approximately 0.36 mg/L corresponding to the applied does of 1.0 mgO_3_/L dose, respectively. Aqueous residual ozone concentrations in the membrane tank were below the level of detection using the indigo method .

The UF permeate was continuously collected by a suction pump at a constant flux of 20 L/(m^2^ h), operated in a cycle of 30 min filtration and 1 min backwash (40 L.m^-2^.h^-1^). For each backwash, air was supplied to each reactor immediately below the membrane modules at 100 L/h (air: water=200:1). The trans-membrane pressure (TMP) was continuously monitored by pressure gauges. The HRT of the membrane tanks was maintained at 0.5 h and accumulated, settled sludge in each tank was released every day. The whole operation process lasted for 70 days. During this period, the membrane only in the CUF-O_3_ system was taken out and washed by sponge at day 30.

**Figure S1** Schematic diagram of the experimental set-up (1 – raw water tank; 2 – feed peristaltic pump; 3 – high level water tank; 4 – constant level water tank; 5 –Al_2_(SO_4_)_3_ tank; 6 – mini-peristaltic pump; 7 – rapid mixing unit; 8– flocculation system; 9 – magnetic stirrer; 10 – CUF tank; 11 – CUF-O_3_ tank; 12 – UF membrane module; 13 – pressure gauge; 14 – suction/backwash peristaltic pump; 15– air/ozone blower; 16 – air flowmeter; 17 – air diffuser; 18 – small air diffuser; 19 – KRC ozone generator; 20 – ozone gas flowmeter; 21 – gas phase ozone analyzer; 22 – ozone destruction unit; 23 – sludge discharge valve).
